# Physiological and Epigenetic Reaction of Barley (*Hordeum vulgare* L.) to the Foliar Application of Silicon under Soil Salinity Conditions

**DOI:** 10.3390/ijms23031149

**Published:** 2022-01-21

**Authors:** Barbara Stadnik, Renata Tobiasz-Salach, Marzena Mazurek

**Affiliations:** 1 Department of Crop Production, University of Rzeszow, Zelwerowicza 4, 35-601 Rzeszow, Poland; rtobiasz@ur.edu.pl; 2 Department of Physiology and Plant Biotechnology, University of Rzeszow, Ćwiklińskiej 2, 35-601 Rzeszow, Poland; marzena.guty@poczta.onet.pl

**Keywords:** chlorophyll fluorescence, gas exchange, methylation-sensitive amplified polymorphism (MSAP), plant stress

## Abstract

Soil salinity is an important environmental factor affecting physiological processes in plants. It is possible to limit the negative effects of salt through the exogenous application of microelements. Silicon (Si) is widely recognized as an element improving plant resistance to abiotic and biotic stresses. The aim of the research was to determine the impact of foliar application of Si on the photosynthetic apparatus, gas exchange and DNA methylation of barley (*Hordeum vulgare* L.) grown under salt stress. Plants grown under controlled pot experiment were exposed to sodium chloride (NaCl) in the soil at a concentration of 200 mM, and two foliar applications of Si were made at three concentrations (0.05%, 0.1% and 0.2%). Measurements were made of relative chlorophyll content in leaves (CCl), gas exchange parameters (C_i,_ E, g_s_, and P_N_), and selected chlorophyll fluorescence parameters (F_v_/F_m_, F_v_/F_0_, PI and RC/ABS). Additionally, DNA methylation level based on cytosine methylation within the 3′CCGG 5′ sequence was analyzed. Salinity had a negative effect on the values of the parameters examined. Exogenous application of Si by spraying leaves increased the values of the measured parameters in plants. Plants treated with NaCl in combination with the moderate (0.1%) and highest (0.2%) dose of Si indicated the lowest methylation level. Decrease of methylation implicated with activation of gene expression resulted in better physiological parameters observed in this group of barley plants.

## 1. Introduction

The primary role of agriculture is to provide food for both humans and animals. The growing world population and the simultaneous constant shrinkage of resources of arable land suitable for food production poses a number of challenges to modern agriculture. At the same time, soils with increased salt content exist in more than 100 countries, and their global area is approximately 1 billion hectares. In total 20% of cultivated land in the world, and 33% of irrigated land, are salt-affected and degraded [1,2,3]. Excessive soil salinity affects the availability and supply of soil nutrients to crops and reduces the productivity, the size and quality of the agricultural crop, which is considered to be one of the world’s most important challenges for agricultural production, food security and sustainability [1,4,5,6]. It is estimated that salinity can reduce yields of all important crops by 20% to 50% [7,8]. The high level of salt in the soil causes two types of stressful situations in plants: osmotic stress and disruption of ion homeostasis [9,10,11]. As a result of osmotic stress, a number of changes occur in plants, leading to an increase in the level of reactive oxygen species (ROS) and the occurrence of oxidative stress [12,13,14,15,16,17]. Plants produce ROS (the singlet oxygen (O_2_), superoxide (O^2−^), hydrogen peroxide (H_2_O_2_) and hydroxyl radical (HO^•^) in chloroplasts, mitochondria, peroxisomes and other sites of the cell because of their metabolic processes such as respiration and photosynthesis [18,19,20]. Photosynthesis is one of the most important processes seriously affected by environmental stress. Abiotic stress causes an excessive reduction in the electron transport chain (ETC), which in turn leads to photooxidation [21]. High salt levels inhibit the activity of the enzymes involved in photosynthesis and has an impact on the proteins involved in both the light and dark phases of photosynthesis. Exposure to salinity also causes a decrease in Rubisco activity and affects CO_2_ binding [22,23,24]. Crop plants induce a complex and unique cellular and molecular response to various stresses to prevent damage and ensure cell survival [6,25,26,27,28,29,30,31]. One of the earliest plant responses to many abiotic stresses is a change in the level of abscisic acid (ABA) [32]. Greater accumulation of ABA promotes a signalling cascade in guard cells, leading to the release of K^+^ ions from guard cells, which results in a reduction in turgor pressure, and then stomatal closure [33,34]. Chlorophyll fluorescence analysis has become one of the most powerful and widely used techniques in plant physiology and is an easy and sensitive method used as an indicator of stress response in plants. It plays an important role in understanding the basic mechanisms of photosynthesis, plant response to environmental change, genetic variability and ecological diversity [35,36,37,38].

Epigenetic mechanisms provide an adaptive layer of control in the regulation of gene expression that enables an organism to adjust to a changing environment. Epigenetic regulation increases the functional complexity of deoxyribonucleic acid (DNA) by altering chromatin structure, nuclear organization, and transcript stability [39]. DNA methylation is known to play an important role in epigenetic mechanisms of the regulation of gene expression in eukaryotes [40]. DNA methylation influences the ability of transcription factors and other DNA-binding proteins to recognize a nucleotide sequence that regulates gene expression. This process is associated with repression of gene transcription [41]. Changes in the methylation patterns of DNA during a cell’s lifetime provide an adaptive ability for the organism to adjust to changes in the environment [39,40,41,42,43].

Methylation-sensitive amplified polymorphism (MSAP) is a powerful technique for studying the genome methylation status. It is a modification of the AFLP technique in which isoschizomers, *Msp*I and *Hpa*II, are employed as ‘frequent-cutter’. Both *Msp*I and *Hpa*II recognize the same restriction site (5′CCGG 3′), but show differential sensitivity to DNA methylation [40,44]. This technique has been applied to study the impact of stress on the level and pattern of DNA methylation in rice [45,46,47], wheat [48], cotton [49] or soybean [50]. Differential DNA methylation patterns and polymorphism among stress-resistant and stress-sensitive plants suggest the possibility of involvement of these distinct DNA fragments in the regulation of physiological processes and morphological traits, enabling adjustment to changing environment conditions [45].

Barley (*Hordeum vulgare* L.) is the most salinity-tolerant species among the cereals, but it reacts negatively at higher concentrations [10]. Due to increasing salinity problems in the world and the negative effects of high salt levels on plants, we must find effective ways to increase the resistance of crop species. The foliar application of micronutrients seems to be the one of the methods of reducing the negative impact of salt stress on plants. Numerous studies conducted on many species of crops have proven the effectiveness of this method of application in creating plant resistance to environmental stresses such as high temperature, drought, salinity, and excess water [51,52,53,54]. The use of micronutrients increases the metabolism of antioxidants in plants [52,55,56,57]. One of the elements that can be used as an alternative method of increasing plant resistance is silicon (Si). Many plants, especially monocotyledons, including barley, contain large amounts of Si—up to 10% dry mass [58,59]. Despite the large accumulation of Si in plants, it has so far not been considered an essential element for higher plants. However, it has a beneficial effect on the growth of many species. Si has a role as a messenger by binding to the hydroxyl groups of proteins involved in cell signalling. Si also has a mechanical effect providing protection to the plant thanks to its deposition in plant tissues [60]. The absorption of toxic ions by the roots is also reduced by Si supply [61]. Many authors have obtained results indicating an important role of silicon in determining the resistance of cereal crops [62,63,64,65,66,67,68]. This element has the ability to strengthen plant defense systems in response to abiotic stresses by increasing the activity of important antioxidant enzymes such as superoxide dismutase (SOD), catalase (CAT) and peroxidase (POD) [56,69,70]. Scientific research shows that silicon is clearly beneficial due to its role in improving the photosynthesis of plants grown under stress conditions [71,72,73,74]. Si can be taken up by both the roots and leaves of plants. Studies prove that foliar application is highly effective, and its positive aspects should also be emphasized—this method of application is cheaper and more convenient to use than soil fertilization. The justification for feeding plants by application of liquid fertilizer containing silicon compounds directly to the leaves also exists because this element is not taken easily by the roots, e.g., in drought or saline conditions [75,76,77,78,79]. Spraying with silicon compounds can have a positive effect on the growth and yield of plants and reduce biotic and abiotic stress, as well as the negative impact of heavy metals on plants [67,80,81,82,83]. Research on silicon and its importance for individual species should be extended due to the number of positive impacts of this element on plants, in particular by increasing resistance to environmental stresses. There are few reports concerning the optimal dose of silicon supplied via foliar application for plants grown under salt stress [84,85,86,87], therefore research was carried out to determine the effect of foliar application of different concentrations of silicon on the activity of the photosynthetic apparatus, gas exchange and the DNA methylation level in barley (*Hordeum vulgare* L.) grown under salt stress conditions. The scientific hypothesis of the study assumes that the foliar application of silicon influences (positively) the response of barley plants at the physiological level to salinity stress. Moreover, the use of different doses of silicon in conditions of salinity stress differentially affects the level of DNA methylation, relative chlorophyll content as well as gas exchange or chlorophyll fluorescence parameters.

## 2. Results

### 2.1. Effect of Salinity and Foliar Application of Si on the Relative Chlorophyll Content of Barley Leaves (CCI)

**Figure 1:**
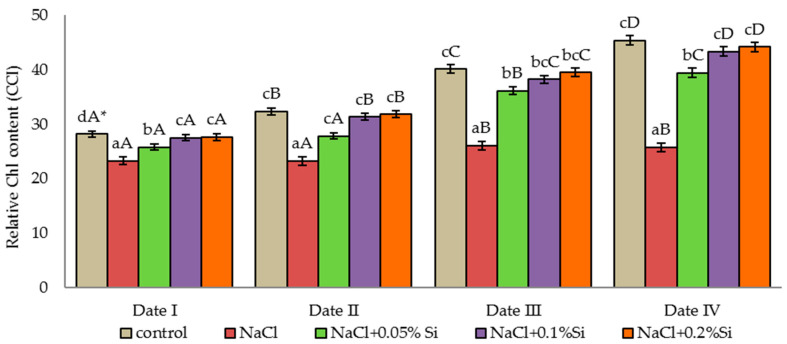
The effect of NaCl, different Si concentration and measurement date on the chlorophyll content in the leaves (CCI); (Date I and Date II, 2 and 7 days after first Si application; Date III and Date IV, 2 and 7 days after second Si application) statistical data are expressed as mean ± SD values. * Capital letters indicate significant differences between the means in the measurement dates for particular Si concentrations, and lowercase letters indicate significant differences between the means at subsequent measurement dates (*p* = 0.05).

The chlorophyll content in the leaves increased with time, regardless of the experimental variant used. For plants grown under saline conditions (without foliar application of Si) the differences in the relative content of chlorophyll between the control plants and the NaCl variant plants increased with time (Figure 1). In the last measurement period (Date IV), 56.8% less chlorophyll was present in the leaves of these plants in comparison with the control plants. The foliar application of silicon in each concentration increased the chlorophyll content in the leaves at each measurement period, compared to the NaCl variant plants, which was statistically confirmed (Figure 1). The use of silicon in a dose of 0.1% and 0.2% caused a similar amount of chlorophyll to be accumulated in the plant leaves as in the control plants, despite their being grown in saline conditions. This relationship proves the positive effect of Si on the accumulation of chlorophyll in barley leaves grown under stressful conditions.

### 2.2. Influence of Salinity and Foliar Application of Si on Gas Exchange Parameters

**Figure 2:**
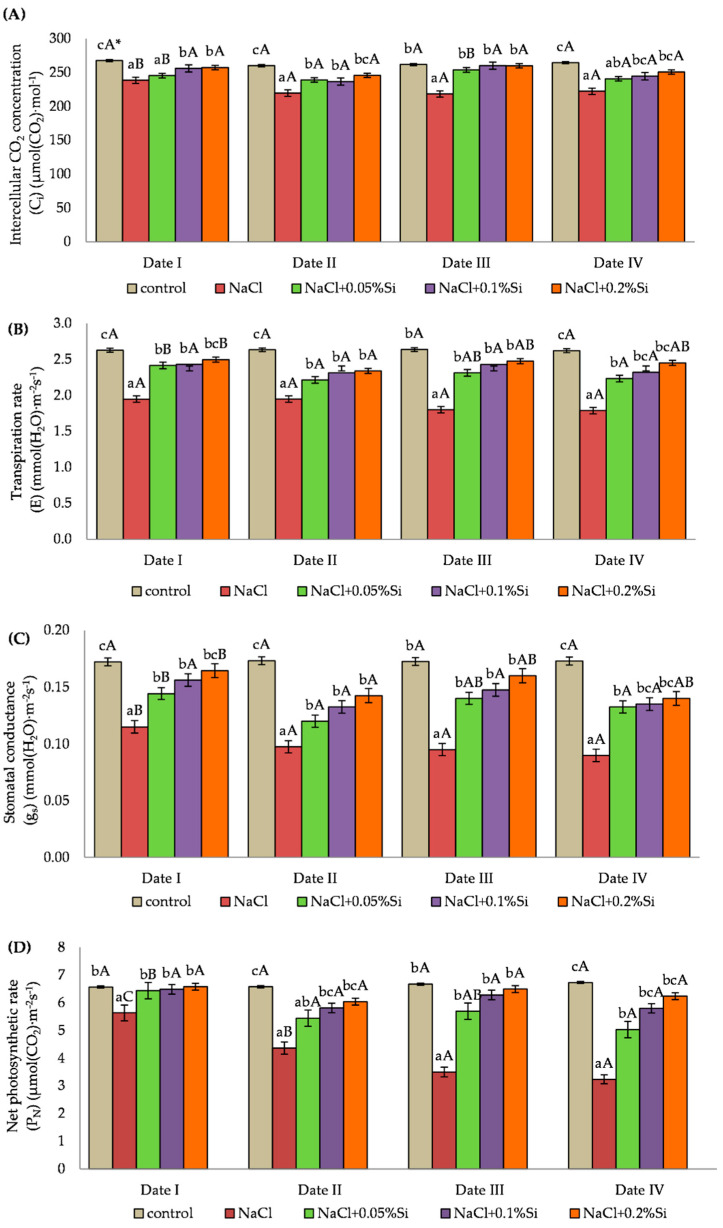
The effect of NaCl, different Si concentration and measurement date on gas exchange parameters: intercellular CO_2_ concentration (C_i_) (**A**), transpiration rate (E) (**B**), stomatal conductance (g_s_) (**C**) and net photosynthetic rate (P_N_) (**D**) in barley plants (Date I and Date II, 2 and 7 days after first Si application; Date III and Date IV, 2 and 7 days after second Si application). Statistical data are expressed as mean ± SD values. * Capital letters indicate significant differences between the means in the measurement dates for particular Si concentrations, and lowercase letters indicate significant differences between the means at subsequent measurement dates (*p* = 0.05).

The C_i_ parameter depended on the applied experience factors (Figure 2A). The lowest values of C_i_ in each of the measurement periods were noted in plants with NaCl without foliar application of Si. After the application of Si, in the first measurement period (two days after the application of the first dose), the plants with 0.1% and 0.2% foliar application of Si had a value of C_i_ 4.5% and 3.8% higher, respectively, in comparison with the control plants. The application of silicon at a level of 0.05% Si caused a statistically significant increase in the measured value of the C_i_ parameter compared to the plants grown under salt stress in the second, third and fourth measurement period. Two days after the application of Si (Date I, III), higher C_i_ values were noted at the same Si concentration when compared to the results obtained on Day 7 (Date II and IV). At the date of the third measurement (two days after the second silicon application), an increased C_i_ was noted in each variant treated with Si, to reach a level comparable with the control plants.

For the barley plants grown under salt stress (without Si) the value of the E transpiration coefficient was reduced by an average of 28.8% when compared to the control plants. The use of silicon, regardless of the concentration, caused an increase of E in comparison with the salt stressed plants in each measurement period. During analysis of the influence of the measurement period on the impact of silicon under saline conditions, higher values were recorded with the concentration of 0.05% Si over the first and third measurement periods (two days after Si application) than over the second and fourth periods (seven days after Si application). The greatest impact of Si on the growth of E in saline conditions was demonstrated at the higher concentrations of 0.1% and 0.2% (Figure 2B).

Stomatal conductance (g_s_) was determined by the experimental factors applied. A decrease by on average 42.5% in the value of the g_s_ parameter in plants grown under salt stress (only NaCl) was noted when compared to the control plants. The use of Si caused an increase in the measured values of the parameter, and the highest values were seen with concentrations of Si of 0.1% and 0.2% in each measurement period when compared to the salt stressed plants (only NaCl). Significant differences in g_s_ values were observed between the measurement periods. The highest values were observed on the second day after Si application (Date I and Date III) (Figure 2C).

Salt stress had a negative effect on the P_N_ index, causing it to decrease. A decrease was observed in the plants with NaCl and the NaCl + Si variant in comparison with the control plants (Figure 2D). The foliar application of Si caused an increase in P_N_ in each measurement period. The highest values were obtained two days after the foliar application of Si (Date I and Date III). In the first measurement period, the use of silicon in plants grown under salt stress caused an increase of the P_N_ index to a value similar to the value of the control plants. In the following periods the P_N_ value decreased when compared to the control plants, but it was still higher when compared to the plants grown under NaCl stress. The duration of the salt stress has an impact on the decrease of the P_N_ value in plants where Si was not applied exogenously. In the fourth measurement period, the P_N_ value was 42.5% lower when compared to the result obtained in the first period. The use of Si in the second measurement period at concentrations of 0.1% and 0.2% increased the P_N_ index by 33.0% and 38.3%, respectively, in comparison with NaCl variant plants. The P_N_ index value with the 0.05% Si concentration on the second day after application (Date I and Date III) was higher than on the seventh day (Date II and Date IV) by an average of 13.7%, which was statistically confirmed.

The values of g_s_ and P_N_ parameters shortly after Si application (two days after spraying) for each Si concentration were similar. Seven days after the application of Si, the significantly highest values were recorded at the concentrations of 0.1% and 0.2% Si. The use of Si in higher concentrations resulted in a longer positive effect on the values of gas exchange parameters compared to the concentration of 0.05% Si (Figure 2C,D).

### 2.3. Influence of Experimental Factors on the Chlorophyll Fluorescence Parameters

**Figure 3:**
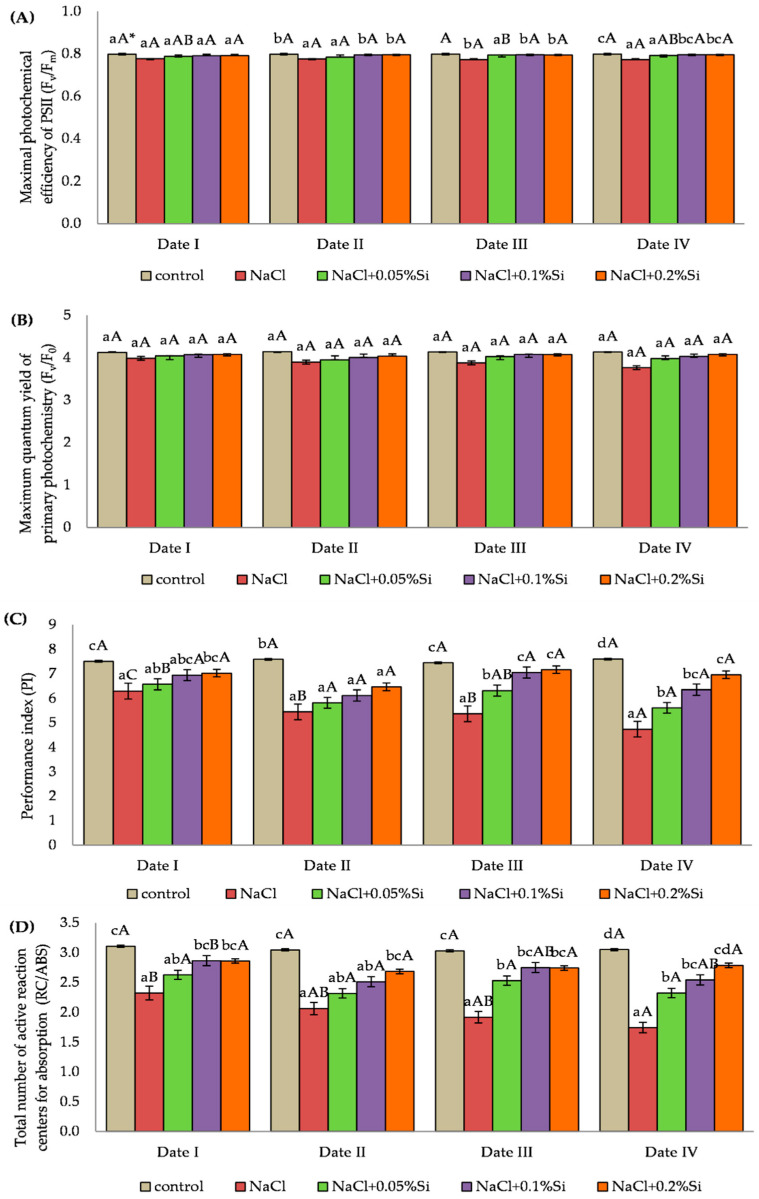
The effect of NaCl, different Si concentration and measurement date on chlorophyll fluorescence parameters: maximal quantum yield of PSII photochemistry (F_v_/F_m_) (**A**), maximum primary photochemistry yield (F_v_/F_0_) (**B**), PS II performance index (PI) (**C**) and total number of active reaction centers for absorption (RC/ABS) (**D**) in barley plants (Date I and Date II, 2 and 7 days after first Si application; Date III and Date IV, 2 and 7 days after second Si application). Statistical data are expressed as mean ± SD values. * Capital letters indicate significant differences between the means in the measurement dates for particular Si concentrations, and lowercase letters indicate significant differences between the means at subsequent measurement dates (*p* = 0.05).

In the first measurement period, salinity and foliar application of silicon had no significant effect on the F_v_/F_m_ value. Lower values of the parameter examined were observed in plants which had an application of salt alone when compared to plants to which silicon had been applied, but these differences were not statistically confirmed. In the next measurement periods of F_v_/F_m_ (Date II, III and IV), the use of silicon at a concentration of 0.1% and 0.2% Si caused a significant increase in F_v_/F_m_ in comparison with plants with NaCl and the NaCl + 0.05% Si variant (Figure 3A).

The analysis of variance did not show any significant influence of salinity and foliar application of silicon on the F_v_/F_0_ value. The plants grown under salt stress (without Si) had lower values of the study parameter when compared to the control plants, but they were not statistically significant. Higher F_v_/F_0_ values were also observed after application of Si over each measurement period compared to the NaCl variant, but this increase was not significant. The highest values, when compared to the control plants, were observed for the plants with NaCl + 0.2% Si variant (Figure 3B).

The PI value depended on both salinity and application of Si (Figure 3C). Plants with NaCl and without foliar application of Si showed a decrease in PI over time. Over the fourth measurement period, the value of the parameter measured in plants with the NaCl variant was 24.7% lower when compared with the first measurement period. Exogenous use of Si caused an increase in the measured value of the parameter. The highest values in salt stressed plants were recorded at the concentrations of 0.1% and 0.2% Si. In plants where silicon was applied, a higher PI value was observed on the second day after application of Si (Date I and III) when compared to the results obtained on the seventh day after foliar application of Si (Date II and IV) (Figure 3C).

Salinity had a negative effect on the RC/ABS value. Plants with NaCl had statistically significant lower values of the measured parameter in comparison with the control plants. Application of Si at each concentration caused an increase in RC/ABS. The highest values in plants grown under salinity stress were observed at the Si concentration of 0.1% and 0.2% (Figure 3D). The analysis of the impact of the measurement period showed a decrease of RC/ABS along with the duration of the stress in plants with the NaCl variant. In the fourth measurement period, the RC/ABS value was 25.0% lower compared to the first period, which was confirmed by the analysis of variance. The highest RC/ABS values in plants grown under salinity stress and silicon application were noted on the second day after application (Date I and III) (Figure 3D).

### 2.4. Effect of Salinity and Foliar Application of Silicon on the Level of DNA Methylation

**Figure 4:**
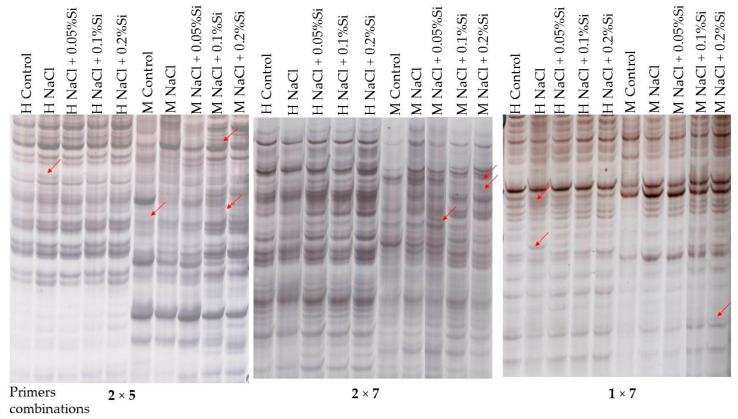
Comparison of representative MSAP gels. H and M refer to digestion with *Eco*RI + *Hpa*II and *Eco*RI + *Msp*I, respectively. Arrows indicate polymorphic bands.

In the presented study, thirteen combinations of selective *Eco*RI and *Msp*I/*Hpa*II primers were used and the numbers of hemimethylated and fully methylated cytosine at 5′CCGG ’3 restriction sites were calculated in barley plants treated with NaCl or in combination with NaCl + Si. DNA methylation profiles with polymorphic fragments occurrence were obtained for an individual analyzed groups of barley plants (Figure 4).

The highest number of DNA fragments were detected in the case of barley plants treated with NaCl in combination with 0.1% and 0.2% Si, whereas the smallest amount of selective amplification DNA products was detected in the case of control barley plants (Table 1). 

However, the number of methylation events was the lowest (17%) in the case of barley plants treated with NaCl and a moderate dose of Si (0.1%) (Table 1). Barley plants treated with NaCl and the highest dose of Si (0.2%) characterized comparably a low level of methylation (19%) (Table 1). In both groups of barley plants, we obtained an almost similar number of PCR products that represented hemi- as well as symmetric methylation of the 5′CCGG 3′ sequence.

Simultaneously, the highest percentage of methylation events was observed in the case of a control group of barley plants, whereas plants treated with NaCl or with a combination with the lowest dose of Si (0.05%) demonstrated a moderate level of methylation events (Table 1). The mentioned group of barley plants characterized a clear advantage of the symmetric methylation of the 5′CCGG 3′ sequence in comparison to hemimethylation. However, in the case of control group plants, the difference was the most distinguished.

## 3. Discussion

Abiotic stresses cause a number of changes in the physiological and biochemical processes as well as on the epigenetic level in plants. Salinity has a negative effect on photosynthesis, which is the most basic and complex physiological process of green plants. Any damage at any stage of the photosynthetic process due to stress can reduce the overall photosynthetic efficiency of plants [22]. The plants in our study were treated with NaCl at a level of 200 mM. Excessive amounts of Na^+^ and Cl^-^ ions in the soil solution induce ionic toxicity by disrupting the structure of enzymes, damaging cell organelles and disrupting cellular metabolism [88,89,90]. In addition, they lead to the accumulation of hazardous substances in plant cells, especially ROS, such as singlet oxygen (O_2_), superoxide radicals (O^2−^) and hydrogen peroxide (H_2_O_2_), which cause oxidative stress and damage proteins, lipids and nucleic acids [91,92]. Energy in the form of ATP triggers biosynthetic reactions in plant cells, which are mainly produced by chloroplasts and mitochondria. When plants are exposed to salinity stress, energy requirements can increase significantly to trigger several energy-intensive adaptive mechanisms ensuring ionic homeostasis, ROS defense and osmotic regulation [93]. Excessive dissipation of photosynthetic energy may have an impact on defensive photo-damage, photoinhibition and photo-oxidative salinity tolerance [94].

Plants are characterized by various levels of salt tolerance [23]. Barley is classified as a species relatively resistant to salinity, but high concentrations of NaCl negatively affect its growth and yield [10]. Chlorophyll is one of the most important features indicating the health condition of plants [95]. The results of the research indicate a negative effect of a high concentration of NaCl in the soil on the relative amount of chlorophyll in leaves and the parameters of plant gas exchange and chlorophyll fluorescence. The chlorophyll content in our study increased over time, but the plants under salinity stress had a lower chlorophyll content when compared to the control plants. A significant decrease in the chlorophyll content in the leaves of plants grown under salinity stress was also noted by other researchers [10,96,97,98,99]. The reduction of photosynthetic pigments may be caused by the breakdown of the thylakoid membranes by the formation of proteolytic enzymes responsible for the degradation of chlorophyll, as well as a result of damage of the photosynthetic apparatus [24,100]. In our research, foliar application of silicon in any concentration increased the chlorophyll content in leaves of barley plants grown under salt stress. Higher values were observed after the use of silicon at concentrations of 0.1% and 0.2% Si than of 0.05%. Similarly, in the studies by Kalteh et al. [101] and studies by Chung et al. [102], the chlorophyll content significantly decreased in response to NaCl and NaCl + Si treatment, and the decrease in the amount of chlorophyll was only greater in plants exposed to NaCl in comparison to control plants. An increase in the chlorophyll content in plants grown under salt stress coupled with an increase in the foliar dose of Si was also noted by Hellal et al. [84] and Abdelaal et al. [57].

In the plants treated with NaCl, there was a significant decrease in the gas exchange parameters P_N_, E, g_s_ and C1. Similarly, in barley grown under saline conditions, a reduction in stomatal conductance (g_s_) and rate of photosynthesis (P_N_) was observed in the studies by Zeehsan et al. [99]. Inhibition of plant assimilation under the influence of salt stress may be caused by a limited supply of CO_2_ due to the partial closure of the stomata and an impaired biochemical binding process for CO_2_ [103]. Plants respond to salt stress by reducing stomatal conductance to avoid excessive water loss [104]. This situation causes the reduction of the photosynthetic binding of CO_2_, a change in the cellular metabolism and an increase in the production of ROS in chloroplasts. ROS can damage the photosynthetic apparatus, especially PSII, causing photoinhibition due to imbalance in the photosynthetic redox signaling pathways and inhibition of PSII repair [105,106,107,108]. Our studies show that the decrease of C_i_ results in a slowing down of the Calvin cycle reaction and the induction of photorespiration. This phenomenon leads to a depletion of oxidized NADP^+^ which acts as a final electron acceptor in PSI and alternatively increases O_2_ electron leakage to form O^2−^ and finally causes the production of more H_2_O_2_ in the peroxisome [15,109]. Lower g_s_ and reduced chlorophyll content may contribute to a greater inhibition of the rate of net photosynthesis (P_N_) [101,110]. The reduction of g_s_, P_N_ and E under the influence of salinity was also noted in studies by other researchers [99,111,112], and salt tolerance depended on the manipulation of the accumulation of E, g_s_ and P_N_, along with increased production of antioxidant enzymes (SOD and POX) at the cellular level. The addition of Si increased the values of the gas exchange parameters that were measured. Similarly, in the studies by Yeo et al. [113], Si induced an increase of g_s_ and P_N_ due to the improved ultrastructural organization of chloroplasts. E, g_s_ and P_N_ also increased under salt stress thanks to the use of silicon in the study by Chung et al. [102] and Li et al. [114]. The protective role of Si in the photosynthetic apparatus, as well as increased photosynthetic activity, can partially be attributed to the greater ability of plants to take up K^+^ and the enhanced antioxidant defense [115]. The increase of gas exchange parameters caused by the foliar application of Si was also noted by De Oliveira et al. [78] in a study conducted on sorghum plants. Rios et al. [116] proposed a model which explained that Si improves the functioning of the stoma and the hydraulic conductivity of the roots by adjustment of aquaporins.

The measurement of chlorophyll fluorescence is considered to be an accurate method that permits the detection of changes in the general bioenergetic conditions of photosynthetic organisms under abiotic stress conditions [117,118,119]. In our studies, the parameters of chlorophyll fluorescence F_v_/F_m_, F_v_/F_0_ and RC/ABS and PI decreased in plants grown under saline conditions, which should be understood as the negative influence of salt on these parameters. The authors of the publications [37,38] indicate that the analysis of chlorophyll fluorescence is useful and accurate as an indicator of plant stress, but in our studies, for the F_v_/F_m_ and F_v_/F_0_ parameters no statistically significant differences were found between the results obtained in the control plants and plants exposed to salinity stress. The negative effect of the salt was limited by the foliar application of Si. According to studies [120,121,122], Si supplied in the form of hydrated rock dust easily and quickly penetrates through the intercellular spaces and stoma of plants, entering into biochemical reactions in cells. When compared to the seventh day, higher values of the gas exchange parameters and chlorophyll fluorescence were observed on the second day after the exogenous application of Si. The main cause of this phenomenon may be the short effect of foliar application of Si. Therefore, the next spray of Si can be treated as an action supporting the positive effect of this element on the plant. According to Laane [81], for most crops, it is optimal to spray the plants 3–4 times during the growing season. The F_v_/F_m_ ratio reflects the photochemical performance of PSII [37]. NaCl stress may disturb the biochemistry of photosynthesis, reducing the efficiency of PSI and PSII due to the disturbance of the integrity of chloroplasts [123], which was demonstrated in our own research, in which a decrease of chlorophyll content was observed in plants grown under saline conditions. The decrease of photochemical efficiency of PSII and F_v_/F_m_ in barley grown under stress was also confirmed by Zeng et al. [96] and Zeehsan et al. [99]. At the same time, the reduction of the RC/ABS ratio as a result of salinity stress in plants was observed by Xia et al. [124] and this phenomenon was also visible in the experiment carried out. The results of our own studies and the literature indicate that increased salinity leads to many changes in energy processes, causing inactivation of reaction centers and inhibition of electron transport, while the foliar application of Si reduces the negative effects of salt in the soil environment.

MSAP analysis confirmed the existence of different levels of DNA methylation among barley plants treated with NaCl or in combination NaCl + Si (Table 1). Barley plants in the control condition (without being NaCl treated) characterized the highest level of DNA methylation. In the case of barley plants treated with NaCl, the total level of methylation decreased. The obtained data indicated the lowest percentage of methylation in the case of barley plants treated with NaCl in combination with moderate (0.1%) and highest (0.2%) dose of Si (Table 1). Decreased methylation level in stress conditions was also proven in tobacco [125], rice [45] and wheat [48]. Moreover, Ferreira et al. [45] indicated that a salt-stress tolerance rice variety characterized a lower level of methylation than a salt stress-sensitive variety. Methylated DNA is well known to inhibit gene expression, while a reduction in the level of methylation leads to an increase of gene expression [126]. Obtained results indicated that in a salt stress condition linked with added NaCl, using more than 0.1% of Si activated more gene expression to cope with stress conditions than without Si or with the smallest dose (0.05%). The decrease of methylation event reflects the properties analyzed on a physiological level (chlorophyll content, gas exchanges, and fluorescence parameters). All of the analyzing features indicated the lowest value in the case of salt stress (NaCl treated). A combined application NaCl with Si (0.1% as well as 0.2%) led to a decrease of methylation (Table 1) and activation of gene expression which probably could result in the increase of chlorophyll level, gas exchange efficiency and chlorophyll fluorescence parameters. However, to confirm this assumption, further analyses, including the investigation expression of specific genes, are required. According to the obtained results, a connection between the ability of DNA methylation adjustment levels and salt stress tolerance can be inferred. Abiotic stresses may cause heritable alterations in cytosine methylation by forming novel epialleles [47,127]. It has been found that plants not only adopt changes for the prevailing stress scenario but also remember this information for the next generation to efficiently cope with such environmental conditions [127]. The memory of a specific environmental stress ability of plants is called ‘plant memory’ or ‘epigenetic memory’. This memory provides a smart basis for a strong and quick response to such future stress challenges [128].

The results of the work indicated differences in the reaction of barley plants on the physiological and epigenetic level in reaction on salt stress. We proved that barley plants which were treated in combination with NaCl and Si (0.1% or 0.2%) indicated higher physiological parameters (chlorophyll content and fluorescence or gas exchange) in comparison to barley plants growing in salt stress condition without adding Si. These results of physiological parameters may be an effect of methylation level reduction. Therefore, it can be inferred that the methylation pattern of barley plants treated with Si is at least partly remembered and inherited. This heritable memory, called ‘plant stress memory’, enables plants to respond against stresses in a better and efficient way, not only for the current plant in prevailing situations but also for future generations. The epigenetic memory of barley plans treated with Si (0.1% as well as 0.2%) will in the future be responsible for appropriate plant reactions to changes to environmental conditions caused, for example, by salinity.

## 4. Materials and Methods

### 4.1. Plant Material and Growing Conditions

A controlled pot experiment was carried out at the University of Rzeszow (Poland). Seeds of the RGT Planet variety of spring barley were sown in 10 cm diameter pots in which was placed 1.5 kg of soil with a grain size of clay sand and a slightly acidic pH (pH: KCl 6.35; H_2_O 6.52). The total content of compounds in the soil: tetraphosphorus decaoxide (P_2_O_5_) 17.4 mg∙100g^−1^, potassium oxide (K_2_O) 17.0 mg∙100g^−1^, magnesium (Mg) 8.87 mg∙100g^−1^, calcium (Ca) 9.46 mg∙100g^−1^. The experiments were carried out in a growth chamber (Model GC-300/1000, JEIO Tech Co., Ltd., Seoul, Korea) at a temperature of 22 ± 2 °C, 60 ± 3% RH, and a photoperiod of 16:8 h light:darkness. The experiment was conducted in a randomized block design with four replicates. The positions of the pots in the experiment were randomly changed every week. 

First, sodium chloride (NaCl) was used as the experimental factor. In the sprout stage of plant growth (the stage of the first pair of leaves), an aqueous solution of NaCl with a concentration of 200 mM in a volume of 50 cm^3^ was applied once to the soil in each pot. The silicon was used twice—after 7 and 14 days from the application of the NaCl solution. Foliar application of Si (200 g1^−1^ SiO_2_) was given in three concentrations of 0.05, 0.1 and 0.2%. Spraying was performed with a laboratory hand sprayer with flow control of a dosing volume of 1.2 mL ± 0.1 during one press (outlet diameter 0.6 mm). This was applied via a uniform spraying procedure. Plants were sprayed until they were dripping. Deionized water was applied at the same time to the control pots. Plants in pots without NaCl (0 mM without addition of NaCl and Si) were used as controls.

Physiological measurements taking place in barley plants were made four times on the first or second fully developed leaves at two- and seven-day intervals after each application of silicon.

### 4.2. Measurement of the Relative Content of Chlorophyll

Two untouched, fully developed leaves in every pot were used to measure the relative chlorophyll content (CCI). Measurement was made using a CCM-200plus hand-held chlorophyll content meter (Opti-Sciences, Hudson, NH, USA). In total, 40 measurements were made per concentration (10 measurements repeated 4 times).

### 4.3. Measurement of Chlorophyll Fluorescence

A continuous excitation Pocket PEA chlorophyll fluorimeter (Pocket PEA, Hansatech Instruments, King’s Lynn, Norfolk, UK) was used to measure chlorophyll fluorescence. This instrument is equipped with clips to darken the leaves which are attached to the leaf blade away from the leaf nerve. The following parameters were measured: maximum quantum yield of PSII photochemistry (F_v_/F_m_), maximum quantum yield of primary photochemistry (F_v_/F_0_), photosynthetic efficiency index (PI) and total number of active reaction centers for absorption (RC/ABS). The fluorescence signal was collected in actinic red light with a peak wavelength of the light source of 627 nm and transmitted for 1 s at the maximum available intensity of 3500 μmol (photon) for photosynthetically active radiation (PAR) m^−2^·s^−1^. Fluorescence measurements were performed three times in each pot on the medial leaf blade after 30 min dark adaptation.

### 4.4. Measurement of Gas Exchange

The LCpro-SD photosynthesis measurement system (ADC Bioscientific Ltd., Herts, UK) was used to measure the photosynthesis of the leaves. The LCpro-SD plat leaf chamber for photosynthesis has a flow accuracy of ± 2% of its range. During the measurement, the light intensity in the chamber was 350 µmol m^−2^∙s^−1^, and the temperature was 23 ± 2 °C. Intercellular CO_2_ concentration (C_i_), transpiration rate (E), stomatal conductance (g_s_) and net photosynthesis rate (P_N_) were measured on two fully developed leaves (*n* = 3).

### 4.5. Methylation-Sensitive Amplification Polymorphism (MSAP) Assay

The materials used in this study included fully expanded leaves of the young seedlings collected on the last day of the experiment. DNA was extracted from fresh leaves of barley plants using the method described by Doyle and Doyle [129]. The MSAP analysis was performed using the protocol described by Xiong et al. [40] and Peraza-Echeverria et al. [44] with some modifications.

Two restriction enzymes, *Hpa*II and *Msp*I, were used to detect cytosine methylation. Both enzymes recognize the tetranucleotide sequence 5′CCGG 3′. The ability to cleave at the recognized sequence is affected by the methylation state of the external or internal cytosine residues. The *Hpa*II is inactive if one or both cytosines are fully methylated (both strands methylated; symmetric methylation) but cleaves the hemimethylated sequence (only one DNA strand methylated), whereas *Msp*I cleaves 5′CmCGG 3′ but not 5′mCCGG 3′ [38,42]. To detect MSAP, two digestion reactions were set up at the same time for each genomic DNA sample. In the first reaction, 0.5 µg of the genomic DNA was digested with 10 U of *Eco*RI (Thermo Scientific, Waltham, MA, USA) plus 10 U of *Msp*I (Thermo Scientific) and 1x Tango buffer (Thermo Scientific) in a final volume of 20 µL for 6 h at 37 °C. The second digestion reaction was carried out as above, however, *Hpa*II (Thermo Scientific) was used instead of *Msp*I. Inactivation were performed at 65 °C for 20 min.

The digestion reactions were then ligated to the adapters by adding 30 µL of ligation mixture containing 1 × T4 DNA ligase buffer (Invitrogen, Waltham, MA, USA), 1 U T4 DNA ligase (Invitrogen), 10 pmol EcoRI adapter (GenoMed, Warsaw, Poland) and 50 pmol *Msp*I-*Hpa*II adapter (GenoMed) (Table 2). The ligation reaction was incubated at 20 °C for overnight. The digestion and ligation reactions were stopped by incubating at 70 °C for 10 min.

The preamplification reaction was performer by using 2.5 µL of the above ligation product with 0.5 µM Pre-*Eco*RI primer (GenoMed) and 0.5 µM Pre-*Msp*I-*Hpa*II primer (GenoMed) with 1× PCR buffer (Dream Taq, Thermo Scientific, Waltham, MA, USA), 1 U Taq polymerase (Dream Taq, Thermo Scientific), 200 µM each of dNTP, in a final volume of 20 µL. The reaction involved 30 cycles of 94 °C for 30 s, 46 °C for 1 min, 72 °C for 1 min, with a final extension at 72 °C for 5 min. Pre-selective PCR products were checked by electrophoresis in 2% agarose gels.

Selective amplification was conducted in volumes of 20 µL. For selective amplification the preamplified mixtures were diluted 10 times from their original volume with 0.1x TE buffer. The amplification reaction was performed by using 5 µL diluted product of preamplification mixed with 0.5 µM of each selective primers (*Eco*RI and of *Msp*I-*Hpa*II, GenoMed) (Table 2), 1 × PCR buffer (Dream Taq, Thermo Scientific), 1 U Taq DNA polymerase (Dream Taq, Thermo Scientific) and 200 µM of each dNTP. Both types of selective primers (*Eco*RI and *Msp*I-*Hpa*II) comprised two or three additional selective oligonucleotides (Table 2). The amplification reactions were performed using the touch-down cycles with the following profile: 12 cycles of 94 °C for 30 s, 65 °C for 1 min reduced by 0.7 °C per cycle and 72 °C for 1 min followed by 24 cycles of 94 °C for 30 s, 56 °C for 1 min, 72 °C for 1 min, with a final extension at 72 °C for 5 min. All amplification reactions were conducted in a thermocycler (Biometra, GmbH, Göttingen, Germany).

A full list of selective primers used in this study are presented in Table 3. 

#### MSAP Electrophoresis and Visualization

The selective PCR products were finally analyzed using 6% denaturing polyacrylamide gel electrophoresis. A mixture of equal volumes of selective PCR products and denaturing formamide dye (98% formamide, 10 mM EDTA pH 8, 0.1% bromophenol blue and 0.1% xylene cyanol) was denatured at 95 °C for 3 min and immediately cooled on ice. Gels were pre-run for 30 min at 60 W first and followed to clean the wells. A 6 µL amount of denatured DNA mixture was loaded in each well and subsequently the gels were run at 60 W for about 2 h. The DNA fragments in gels were stained via the silver staining method following Bassam and Gresshoff [130]. The 100 bp ladder (BioTools, Inc., Jupiter, FL, USA) was used as a molecular size marker. The reproducibility of the methylation patterns was confirmed by repeating the experiments twice.

### 4.6. Methylation Analysis

According to information presented by Xiong et al. [40] and Walder [131] a DNA methylation event was detected when bands present in the gel from the reaction *Eco*RI + *Msp*I (M) were absent from the reaction *Eco*RI + *Hpa*II (H). This indicated that the internal cytosine was methylated (5′C^m^CGG 3′). This is regarded as the ‘symmetric or full methylation’. The contrary situation, where a band was present in H but absent in M, indicated that the external cytosine of one DNA strand was methylated (5′^m^CCGG 3′). This is regarded as the ‘hemimethylated state’. Percentage methylation was calculated followed Xiangqiana et al. [132] as below:

Methylation (%) = (number of methylated bands/total number of bands) × 100

### 4.7. Statistical Analysis

Statistical analysis was performed using TIBCO Statistica 13.3.0 (TIBCO Software Inc., Palo Alto, CA, USA). In order to detect all departures from a normal distribution at *p* = 0.05, the Shapiro–Wilk test was performed. The homogeneity of variance was checked. Two-way repeated measures ANOVA was then performed (with time of assessment as a factor). In order to determine and verify the relationship, Tukey’s post hoc test was performed with a significance level *p* ≤ 0.05.

## 5. Conclusions

The aim of the studies was to evaluate the effect of foliar application of Si on the photosynthetic apparatus, gas exchange and methylation level of barley *(Hordeum vulgare* L.) grown under salt stress. On the basis of the research conducted, a positive effect of Si was demonstrated on the relative content of chlorophyll in leaves and the selected parameters of chlorophyll fluorescence and gas exchange in plants. The dose of 0.2% Si turned out to be the most beneficial for barley plants grown under salt stress. The highest content of chlorophyll CCI in the leaves was reached during its application. These plants had higher gas exchange parameters (C_i_, E, g_s_, P_N_) and chlorophyll fluorescence parameters (F_v_/F_0_, F_v_/F_m_, PI and RC/ABS) when compared to the Si dose of 0.05% and 0.1%. The action of silicon at the level of 0.05% Si was more effective immediately after the foliar spraying of plants. Higher values were recorded on the second day after application in comparison with the seventh day after application.

The MSAP analysis confirmed the existence of different levels of DNA methylation in barley plants grown under saline soil conditions. The highest level of DNA methylation was found in barley control plants. Salt stress, as an effect of NaCl, caused a decrease in DNA methylation in barley plants. The lowest level of DNA methylation was observed in barley plants treated with NaCl in combination with a moderate (0.1%) dose of Si. It is a well-known fact that the decreased methylation leads to an increase in gene expression. The decrease in the methylation level of barley plants treated with the NaCl + Si mixture reflects the observed physiological properties (chlorophyll content, gas exchange, fluorescence parameters). Epigenetic changes induced by salinity stress trigger a ‘plant epigenetic stress memory’ that enables plants treated with Si to respond to stress in a better and more effective manner, not only for the present plants but also for future generations.

The conducted research confirmed the hypothesis that silicon foliar spraying influences (positively) the response of barley plants at the physiological level to salinity stress. Moreover, the use of different doses of silicon in conditions of salinity stress differentially affects the level of DNA methylation, relative chlorophyll content as well as gas exchange or chlorophyll fluorescence parameters. The obtained results can be used as a basis for the development of a strategy for reducing the negative impact of abiotic stresses on agricultural productivity. Foliar application of silicon can be an effective and environmentally friendly method of reducing the impact of soil salinity on crops. The application of Si in saline soil conditions initiates the development of resistance to stress in barley plants and may lead to an increase in yield potential and stability in the future. In order to confirm the results obtained, further analyses should be performed for other plant species. Simultaneously, an extended molecular multivariate analysis confirming that the use of Si leads to a decrease of methylation together with activation expression of specified genes should be performed. The obtained test results should be verified in the field because the course of weather conditions can modify the reaction of plants to stress conditions, especially during plant vegetative growth, which is one of the most important factors determining the growth and yield of crops.

## Figures and Tables

**Table 1:** Summary of DNA methylation level.

Analyzed Values	Control	NaCl	NaCl + 0.05% Si	NaCl + 0.1% Si	NaCl+ 0.2% Si
Total bands number	858	878	895	952	938
Number of symmetric methylation bands	159	114	137	80	85
Symmetric methylation (%)	19%	13%	15%	8%	9%
Number of hemimethylation bands	89	90	78	78	91
Hemimethylation bands (%)	10%	10%	9%	8%	10%
% total methylation	29%	23%	24%	17%	19%

**Table 2:** Sequences of adapters and primers used for MSAP analysis.

MSAP Stage	Primers/Adapters	Sequences
Ligation	*Eco*RI-Adapter	5′CTCGTAGACTGCGTACC 3′ 3′CATCTGACGCATGGTTAA 5′
	*Msp*I-*Hpa*II-Adapter	5‘CGACTCAGGACTCAT 3′ 3′TGAGTCCTGAGTAGCAG 5′
Preamplification	Pre-*Eco*RI	5′GACTGCGTACCAATTC 3′
	Pre-*Msp*I-*Hpa*II	5′GATGAGTCCTGAGTCGG 3′

**Table 3:** Sequences of primers used for selective amplification.

No.	Primer Name	Sequences
0	*Eco*RI-ACT	5′GACTGCGTACCAATTCACT 3′
1	*Eco*RI-AG	5′GACTGCGTACCAATTCAG 3′
2	*Eco*RI-AC	5′GACTGCGTACCAATTCAC 3′
3	*Eco*RI-AT	5′GACTGCGTACCAATTCAT 3′
4	*Msp*I/*Hpa*II-ATG	5′GATGAGTCCTGAGTCGGATG 3′
5	*Msp*I/*Hpa*II-CTA	5′GATGAGTCCTGAGTCGGCTA 3′
6	*Msp*I/*Hpa*II-CTC	5′GATGAGTCCTGAGTCGGCTC 3′
7	*Msp*I/*Hpa*II-CAT	5′GATGAGTCCTGAGTCGGCAT 3′
8	*Msp*I/*Hpa*II-CT	5′GATGAGTCCTGAGTCGGCT 3′
9	*Msp*I/*Hpa*II-GT	5′GATGAGTCCTGAGTCGGGT 3′
10	*Msp*I/*Hpa*II-CA	5′GATGAGTCCTGAGTCGGCA 3′

## Data Availability

The data presented in this study are available upon request from the corresponding author upon reasonable request.
